# Optical absorbance of the tympanic membrane in rat and human samples

**DOI:** 10.1371/journal.pone.0254902

**Published:** 2021-07-22

**Authors:** Madeleine Goblet, Farnaz Matin, Thomas Lenarz, Gerrit Paasche

**Affiliations:** 1 Department of Otorhinolaryngology, Hannover Medical School, Hannover, Germany; 2 Hearing4all Cluster of Excellence, Hannover Medical School, Hannover, Germany; Massachusetts General Hospital, UNITED STATES

## Abstract

Chronic infections are often connected to biofilm formation. In presence of implants, this can lead to loss of the implant. Systemic or local application of drugs is relatively ineffective in case of biofilm formation. One technique to provide antibacterial properties on demand is the antibacterial photodynamic therapy (aPDT). Using this technique, these properties can be “switched on” by light illumination. In the middle ear with the semitransparent tympanic membrane, it might be possible in future to activate the antibacterial effect without opening the membrane. Therefore, we investigated the optical absorbance spectra of the tympanic membrane. Optical absorbance spectra were measured in *ex vivo* preparations from neonatal and adult rats with the membrane still being attached to the surrounding bony ring and four human samples. After performing area scans, the spot with the lowest absorbance being surrounded by a ring like structure with higher absorbance was chosen as region of interest for scanning wavelengths between 300 and 900 nm. Absorbance is generally higher at lower wavelengths with a local absorbance maximum at 420 nm and a weak second maximum with two neighbouring peaks at 540 / 580 nm and is significantly higher in adult rats compared to neonatal rats where about 10% of light was transmitted. The human samples show similar characteristics with a little higher absorbance. For activation of aPDT through the tympanic membrane, larger wavelengths are more promising. Whether the amount of light transmitted through the membrane would be sufficient to induce aPDT remains to be tested in further experiments.

## Introduction

One of the key components for sound transmission to the auditory system is the tympanic membrane (TM) which separates the external ear from the middle ear [1, 2]. Acoustic sound waves are transformed into mechanical vibrations and transmitted from the outer ear canal to the ossicles of the middle ear [1, 3]. The human TM is a tri-laminar membrane with a thin outer epidermal layer (lateral side), a middle *lamina propria* (intermediate fibrous layer) and a very thin inner mucosal epithelial layer (medial side) of cells. The overall membrane thickness is about 100 μm [4], non-uniform and tapered from periphery to the center of the TM, the umbo [2, 3]. The particular orientation of the TM enables a larger surface than the outer ear canal diameter [2]. It is almost oval in shape and conical in cross section, with the apex pointing medially towards the middle ear [3]. The boundary of the TM is strongly anchored to the wall of the tympanic cavity. Most of its boundary is formed by a fibro-cartilaginous ring called the annulus. This ligament is a fibrous thickening firmly attached to a sulcus in the bony tympanic ring except superiorly where it separates the two main regions or sub-membranes of the TM called the pars tensa (PT) and the pars flaccida (PF) [2, 3]. The PF is located in the superior region and is more fragile as it lacks the fibrous middle layer of the TM [1, 3]. The function of the PF remains unclear. The PT covers most of the TM and is attached to the manubrium of the malleus and directly responsible for transmitting sound from the ear canal to the middle ear ossicles. The manubrium stretches from a point on the superior edge of the TM to the umbo [2, 3]. Changes in structure and mechanical properties of the TM due to middle ear diseases, such as middle ear infection, otitis media with effusion, or perforation of the TM can deteriorate sound transmission and cause conductive hearing loss [3]. One of the most common infections of the middle ear is otitis media (OM). Three different types of OM are described, which are closely related and symptoms can overlap: acute OM (AOM), OM with effusion (OME) and chronic suppurative OM (CSOM) [5, 6]. Inflammation of the middle ear can produce excess fluid behind the TM, and OME is essentially characterized by fluid accumulation in the middle ear cavity behind an intact TM [7, 8]. This fluid accumulation in the middle ear can be detected by otoscopy through the intact semitransparent TM [9].

Repeated acute infections that persist more than three months can become chronic and chronic infections are often connected to the formation of biofilm. This is especially important when implants are present at the location of the infection because it can lead to loss of the implant. In an investigation of 15 explanted cochlear implants bacterial colonization was found on eight implants [10]. In three out of four devices explanted due to infections, a bacterial colonization was found. Most common germs in the investigation were *Staphylococcus aureus*, *Pseudomonas aeruginosa*, and *Haemophilus influenza* [10]. Other authors report that implants can repeatedly evoke inflammation in the surrounding of the implant, whereby in 65% of chronic infections biofilm formation is involved [11].

Biofilm can contain bacterial or fungal cells that are in contact with each other. The cells are trapped within an adherent matrix on an inert or living surface. The extracellular matrix protects the bacteria against antibodies, phagocytosis and antibiotics [11]. Therefore, systemic or local application of antibiotic drugs is relatively ineffective in case of biofilm formation [12]. To prevent biofilm formation especially on implant surfaces, it is possible to add drug-containing coatings to the implants. Drug-releasing coatings are clinically used for different purposes [13]. But as the coating serves as reservoir for the drugs, the amount of drug that can be stored is limited [14]. That is why it would be more beneficial to have a drug-release on demand. One technique that provides antibacterial properties on demand is the antibacterial photodynamic therapy (aPDT) [15]. Here, antibacterial properties can be “switched on” by illumination with light [16]. So far, aPDT is mainly tested and used for dental applications [17, 18] but also wound healing [19]. Additionally, few reports on application of aPDT on common bacteria causing OME in vivo [20] and in vitro [15] are available. In the case of the in vivo study, the fiber for light activation was inserted into the bullae through the tympanic membrane of gerbils [20].

As more aPDT substances are emerging, it might be possible in future to activate the antibacterial effect without opening the TM as the TM is semitransparent. Therefore, the aim of the study was to investigate light transmission properties (absorbance) of the TM. This was done in *ex vivo* preparations of the TM from young and adult rats, and additionally, four human TM could be tested.

## Material and methods

### Ethical statement

The experiments were accomplished in accordance with the German “Law on Protecting Animals” (§4) and the European Directive 2010/63/EU for protection of animals used for experimental purpose, and registered (neonatal rat: no. 2016/118; adult rat: no. 2016/125) with the local authorities (Lower Saxony State Office for Consumer Protection and Food Safety (LAVES), Oldenburg, Germany).

The collection and use of human tympanic membranes was approved by the institutional ethical committee of Hannover Medical School and registered under number 8375_BO_K_2019. Written informed consent was obtained from all patients. All personal information that could lead to identification was removed.

### Preparation of neonatal rat tympanic membranes

**Fig 1:**
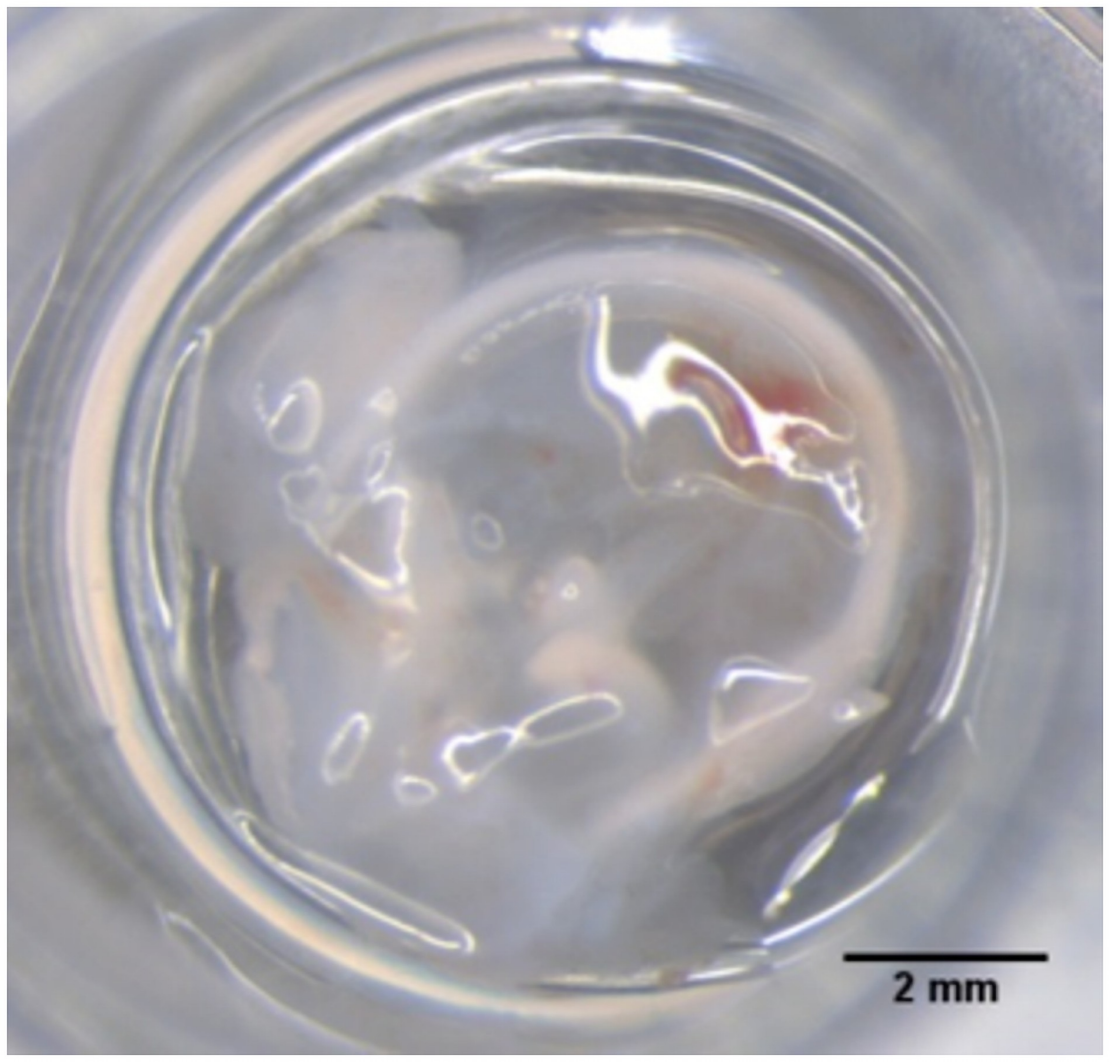
Tympanic membrane (rat P3-5). The TM was positioned centrally on the lid of a 96-well plate for measurements.

The absorbance of tympanic membranes was analyzed with freshly isolated samples. Neonatal Sprague-Dawley rats (p3-5) of different sexes were used for dissection of the tympanic membrane from the petrous bone. After rapid decapitation the skull was opened along the midline, removing the brain, and separating it into two halves, according to the previously described protocol for isolation of spiral ganglion neurons [21]. The head halves were transferred into ice-cold phosphate-buffered saline (PBS; PBS tablets, Gibco^®^ Thermo Fisher Scientific, Waltham, US). All further preparation of the TM took place under microscopic magnification (Leica M165 C, Bensheim, Germany). The surrounding tissue and ossicles were removed carefully with fine forceps and the tympanic membrane remained attached to the surrounding bony ring. The TM was then placed on the lid of a 96-well plate (TPP, Trasadingen, Switzerland) for the measurements (Fig 1).

### Preparation of adult rat tympanic membranes

Fresh cadavers of adult Sprague-Dawley rats (age between 6 months and 2 years) were received from the central animal facility. The skull was removed with a scissor and the fur was retracted until the ears were free. Muscles and tissue around the outer ear canal were removed and the petrous bone was detached from the skull with Luer bone pliers. The area of the TM was cleaned as good as possible from ossicles, muscles and other tissue without damaging the membrane. Finally the TM was placed centrally on the pit of a 96-multiwell culture plate lid with the outer ear canal side facing the lid for absorbance measurements.

### Human tympanic membrane

In this study, samples of four human TM were measured for comparison. The TM were harvested from tissue samples that are usually disposed due to temporal bone surgery with complete exenteration of all pneumatic tracts also termed as subtotal petrosectomy (STP) with obliteration of the middle ear and mastoid and closure of the external auditory canal. An overview on patients is provided in Table 1.

**Table 1:** Patient information for human samples.

Sample	Gender	Age	Etiology
**1**	female	68y	s/a cochlear implantation and subsequent electrode dislocation in the mastoid with a prolapse of the electrode array into the outer ear canal (right side)
**2**	female	63y	s/a deafness on both sides after skull base fracture in childhood, s/a multiple ear surgerys with open mastoid cavity (so called radical cavity) on both sides
**3**	male	56y	s/a radical cavity after recurrent cholesteatoma right, surditas right
**4**	male	62y	s/a extensive cholesteatoma in the mastoid left, surditas left

**Fig 2:**
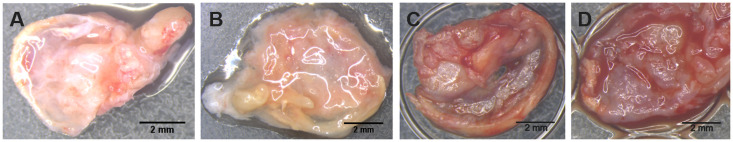
Human tympanic membranes after preparation. (**A**) Tympanic membrane with attached malleus cleaned as good as possible before measurements. (**B-D**) Tympanic membranes as received from the OR without malleus.

After intraoperative removal of the TM these were stored in PBS for immediate transfer to the lab. The samples were carefully cleaned by removing surrounding tissue with fine forceps. In one case the malleus was not removed from the TM (Fig 2A) because of an expected high risk of damage to the TM. For measurements, samples were also placed centrally on the lid of a 96-multiwell plate (TPP) with the ear canal side facing the lid (Fig 2A–2D).

### Experimental setting for absorbance measurements

Absorbance measurements of the TM were performed at wavelengths between 300 and 900 nm by using a plate reader (Synergy H1 Hybrid Reader; BioTek, Bad Friedrichshall, Germany). The finally prepared TM’s were placed centrally on the pit of a 96-multiwell culture plate lid (TPP) for absorbance measurements. First, a spectral scan was performed. For this, the system used one spot per well and measured the transmitted light in the defined range of wavelengths in 10 nm steps. In a second measurement, an area scan with 81 measuring points (9x9 matrix) and 20 nm steps over the wavelength range was performed. As internal control the wavelength 300 nm was measured again at the end of the area scan. In a third measurement, a second spectral scan was performed as additional control. For each series of measurements n = 6–8 tympanic membranes were used. In addition to the TM’s also controls in empty and covered wells were measured. For covering the wells, paper towels or bone were used allowing at least some light passing through. Completely intransparent materials (paper wrapped grey plastic) were used for a complete block of the light pass.

### Data evaluation

Absorbance (*A*) is calculated from *A = log*_*10*_ *I*_*0*_*/I* with *I*_*0*_ being the initial light intensity and *I* the transmitted intensity and is therefore dimensionless. An absorbance of 0 means that 100% of light is transmitted, absorbance of 1 stands for transmission of 10% of the initial light intensity and so on. The quotient *I/I*_*0*_ is also called transmittance.

**Fig 3:**
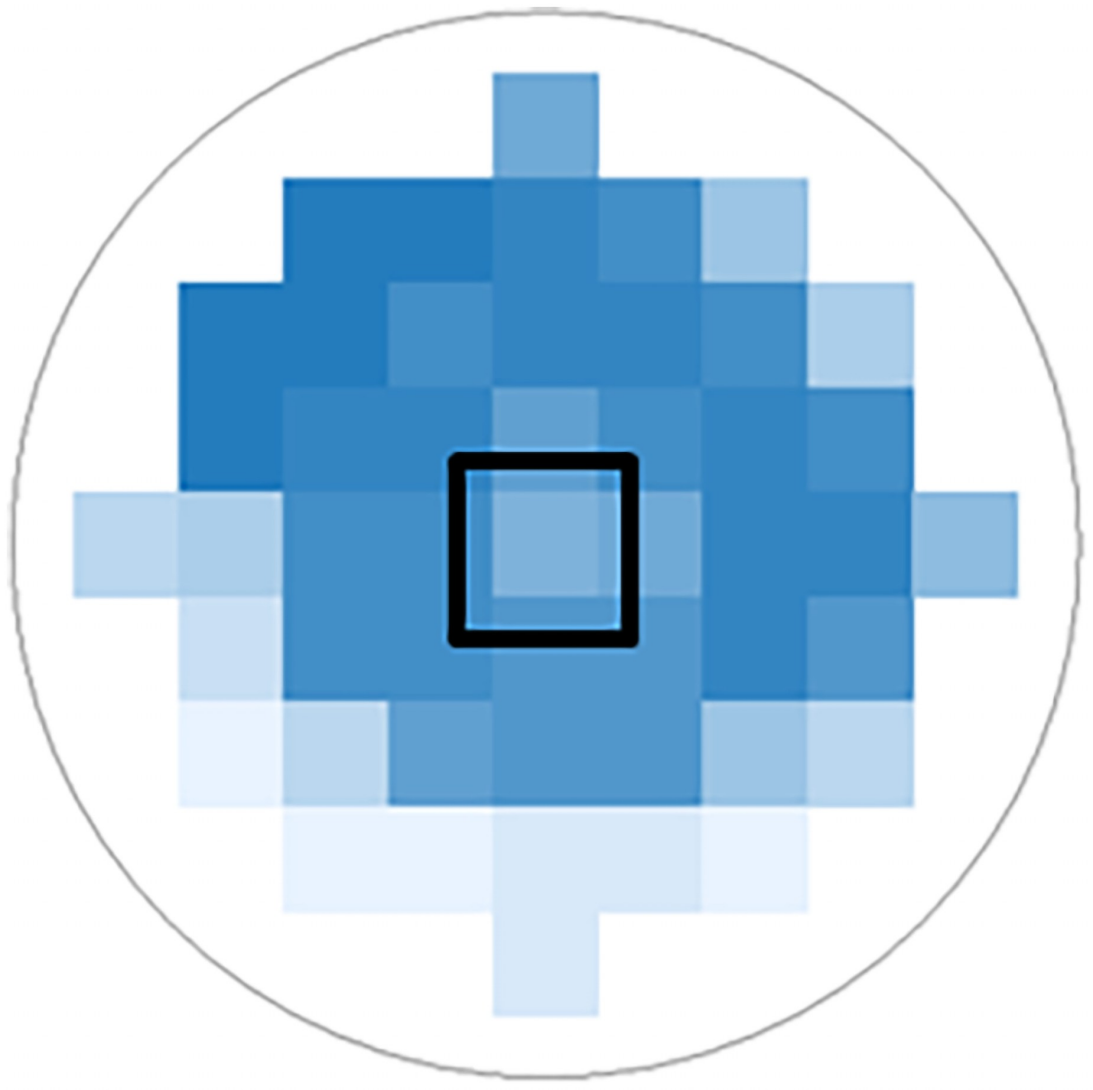
Heat map of absorbance measurements of a tympanic membrane at 300 nm with a 9x9 matrix. The point with the least absorbance (ROI) was marked in black. Spots with no light transmission are presented as white areas in the heat map.

To evaluate the absorbance at different wavelengths of a scan the software of the system calculates one absorbance value for each measured wavelength at each spot. Having performed the area scans, the system generates a heat map for each measured wavelength (compare Fig 3). Light blue stands for high transmission (low absorbance, low optical density (OD)), dark blue for low transmission (high absorbance, high OD). If the system did not detect transmitted light at a single spot, these areas were left white. In the heat map generated for the wavelength of 300 nm, the spot with lowest absorbance (marked black in the example of Fig 3), being surrounded by a ring like region with higher absorbance, was chosen as region of interest (ROI). The absorbance at this spot was taken as value for the absorbance of the TM. To evaluate the absorbance at different wavelengths, the defined ROI was transferred to all other heat maps of the same TM and the referring absorbance values were collected accordingly.

### Statistical analysis

Statistical analyses were performed using GraphPad Prism version 5.02 for Windows (GraphPad, La Jolla, California, US) and OriginPro 2021 (OriginLab Corp., Northampton, MA, US). Different scans were compared by 2-way ANOVA (repeated measures) whereas drying of TMs was evaluated by paired t-test.

## Results

### Rat tympanic membrane

**Fig 4:**
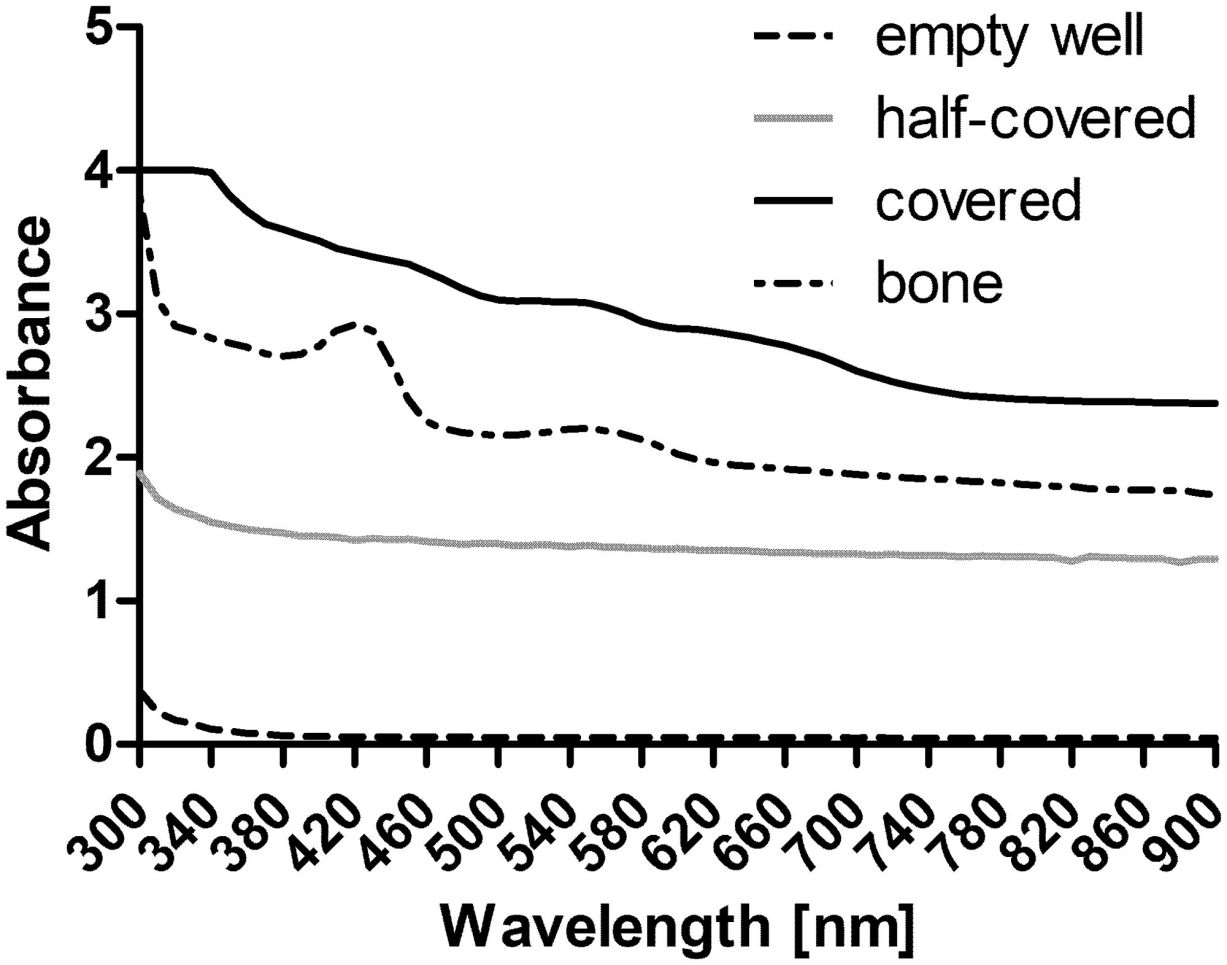
Spectral scan of control wells from 300 nm to 900 nm. To determine the upper and lower limits, wells were covered (black line) or partly covered (grey line) with paper or the bottom of the well was covered with bone (dotted line). For comparison a spectrum of an empty well (dashed line) is provided.

In a first step, upper and lower limits were determined by covering the wells with paper towels or bone (Fig 4). An absorbance of approximately zero was measured with empty wells (black dashed line), and an absorbance between 2.5 and 4 was measured with paper towels or bone (black or dotted line). Data of completely covered wells are not shown due to full absorbance (transmittance of zero; infinite absorbance).

**Fig 5:**
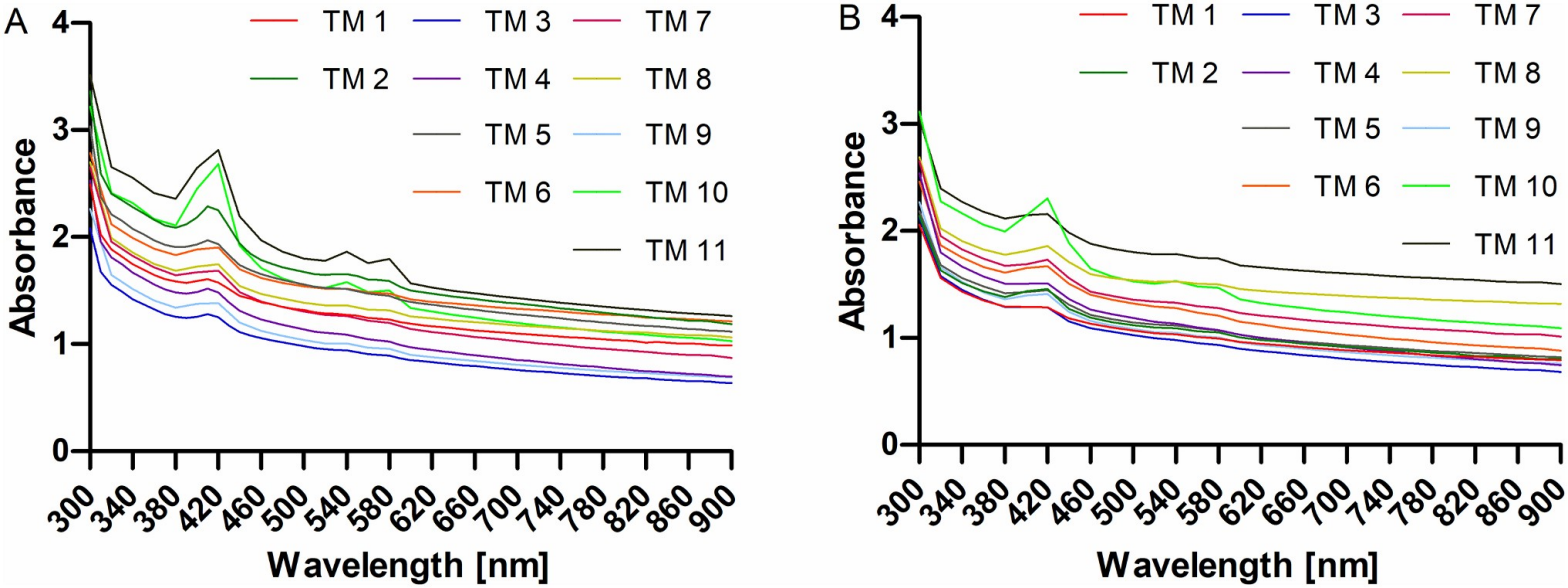
Comparison of spectral scan (A) and area scan (B) of 11 TM from 300 nm to 900 nm.

Scans of individual TM’s of neonatal rats are provided in Fig 5 as spectral scan and area scan. The spectral scan measurements (Fig 5A) are based on one large spot per well, it contains information from bone, TM, tissue, but also empty areas. To investigate the TM more specifically, area scans (Fig 5B) were performed as described above. Both scans show comparable characteristics over the tested wavelengths (300–900 nm). Absorbance is generally higher at lower wavelengths and appears to be slightly lower in the area scan compared to the spectral scan. The difference between average values of both scans is significant for wavelengths 300 nm to 460 nm (2-way ANOVA). In all scans, a local absorbance maximum was detected at 420 nm. A weak second maximum with two neighboring peaks was found at 540 nm / 580 nm. Generally, these maxima are less pronounced in the area scans.

**Fig 6:**
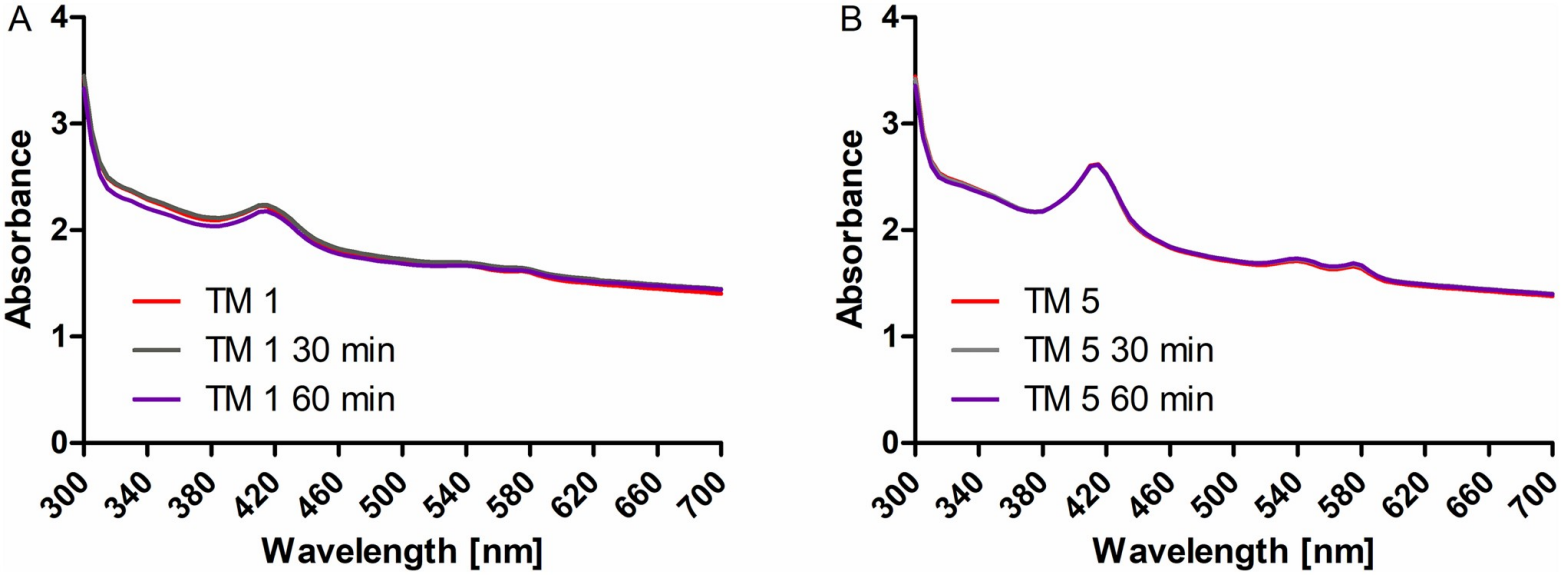
Spectral scan of two TM directly after preparation (red), after 30 min (grey) and after 60 min (purple). No effects of drying were detected without additional wetting for at least 1 hour.

To investigate whether results were influenced by drying of the TM during the measurements and samples should be wetted, 100 μl PBS were added to one sample before the scan. During the area scan, absorbance of this TM increased between measurements at 500 nm and 520 nm from 1.15 to 2.09. As this was interpreted as movement of the sample in the well, all other reported measurements were done without addition of fluid. To test for the possible influence of drying in this approach, for each TM, measurements at 300 nm were done first and repeated at the end of the different scans. Paired t-tests revealed no difference between measured absorbance (p = 0.471). In addition, for some TM spectral scans (300 nm to 700 nm) were repeated after 30 min and 60 min (Fig 6). No differences compared to the initial scans were detected. For other TM (N = 5), area scans (300 nm to 900 nm) were repeated after 2 hours. Also in these cases no differences to the initial scans were found (S1 Fig).

**Fig 7:**
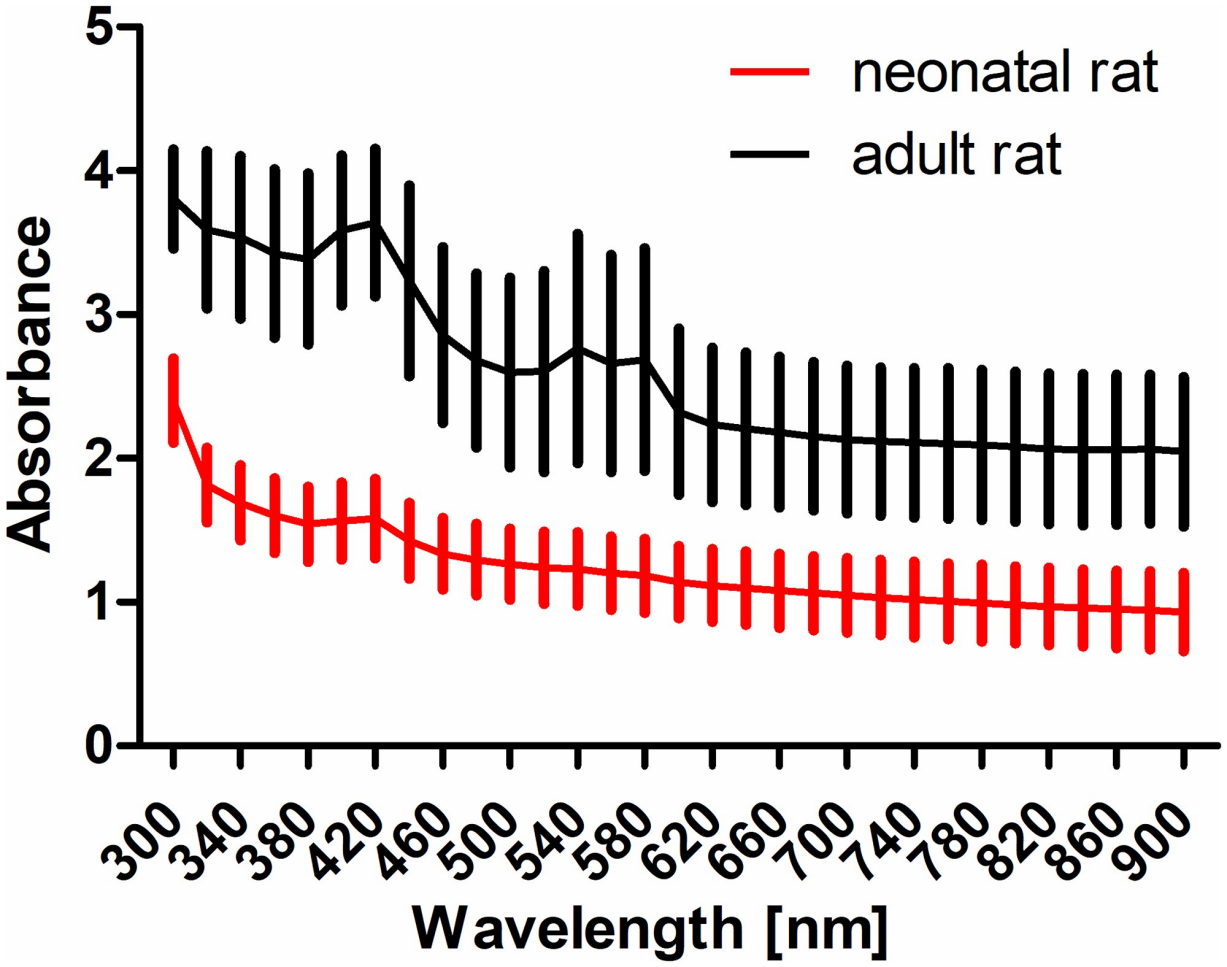
Absorbance of TM from neonatal and adult rats. All TM were measured from 300 nm to 900 nm in area scan mode. Presented are mean values ± standard deviations.

All measurements presented so far were performed with TMs from neonatal rats. As the auditory organ in neonatal rats is still immature [22], measurements were also performed using TMs from adult rats. A comparison of mean absorbance values as received using the area scan approach is provided in Fig 7. Absorbance of the adult TM is significantly higher at all measured wavelengths (p < 0.001 at all wavelengths). In the wavelength range from 800 to 900 nm the amount of transmitted light is slightly above 10% in neonatal TM and gets reduced to about 1% in adult rats.

### Human tympanic membrane

**Fig 8:**
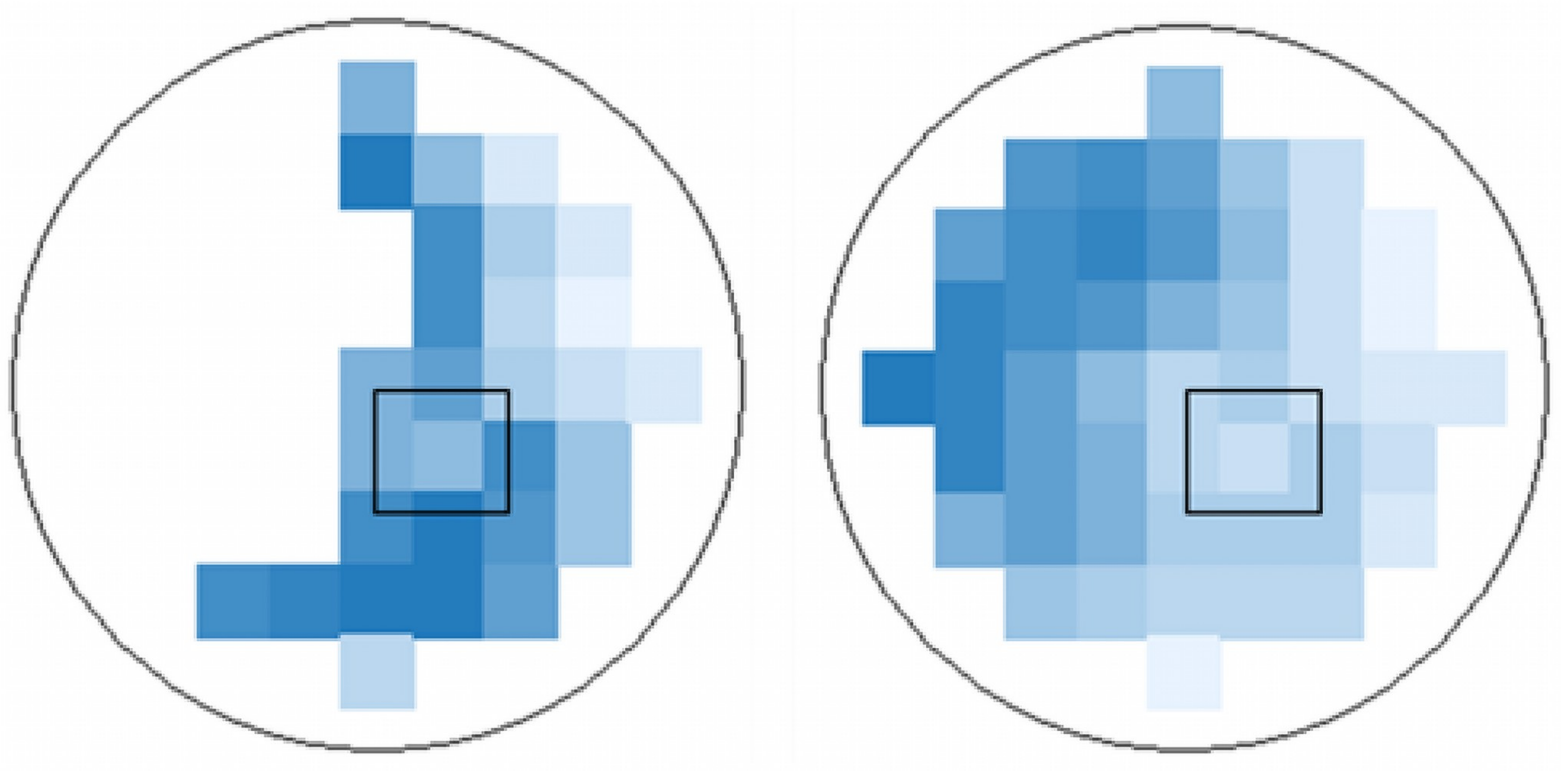
Heat map (9x9 matrix) of sample 1 at 380 nm (left) and 460 nm (right). The point with the least absorption (ROI) is marked in black. No light was transmitted at lower wavelengths in large parts of the sample.

**Fig 9:**
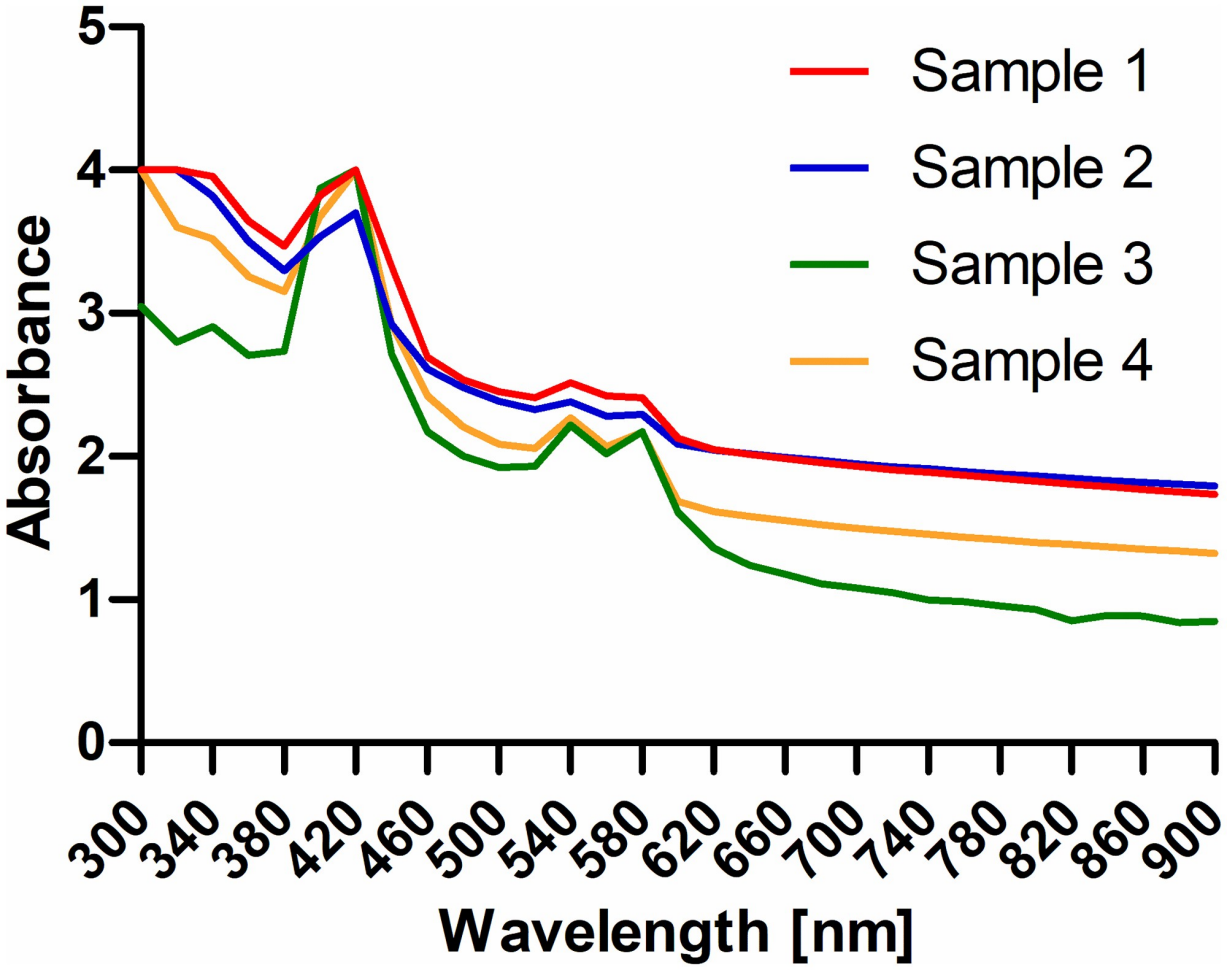
Area scan of four human TM samples. Absorbance was measured at the spot of least absorption at wavelengths between 300 nm and 900 nm.

The measuring system established with the rat samples was also used for four human samples. To find the spot with the least absorbance (ROI) it was necessary to use the 460 nm 9x9 matrix because in a large part of the sample no light was transmitted at lower wavelengths (Fig 8). Finally all scans show the same characteristics as the rat TMs but with little higher absorbance (Fig 9). Variability between the scans appears to be larger compared to the rat results especially with regard to the characteristic peaks. The position of the peaks was not changed compared to the rat TM. Before and after each area scan, spectral scans were performed to control for time dependent changes such as drying. Only with sample three some changes in spectral characteristics were detected. Here, absorbance was reduced to values below 1 at all wavelengths above 300 nm (S2 Fig).

## Discussion

Implant associated infections can lead to biofilm formation and are one of the main reasons for complications after implantation. In the worst case, it can lead to explantation of the affected device. Therefore antibacterial photodynamic therapy (aPDT) can be a good solution to eliminate the resistant pathogens that cannot be managed by antibiotics [20]. aPDT is based on photosensitizers (PSs) activated by light at PS-specific wavelengths (visible or ultraviolet light). Activation of a PS leads to generation of singlet oxygen and other reactive oxygen species (ROS). The production of ROS induces irreparable and lethal oxidative damage affecting the integrity of the cell surface and intracellular bacterial biomolecules as well as significant destruction and disintegration of the biofilm matrix [15, 16, 20]. aPDT can also be considered as an alternative therapy in selective cases of OM because the middle ear cavity provides space, which can store the PS for a given time period [20]. In the mentioned study, the aPDT effect was initiated by a 632 nm diode laser with the tip of the fiber being advanced through the TM into the bulla of gerbils. As the TM is semitransparent, it might also be possible to activate the effect by bringing the light source in front of the TM without opening it. To investigate this possibility it is necessary to know the absorbance spectra of the TM.

The TM works optically like a translucent diffuser but reflects a small amount of light [8]. Reflection characteristics were earlier investigated by different authors [4, 23]. For human TM, reflection characteristics were investigated for wavelengths from 400 nm to 900 nm in cadavers with and without contributions of middle ear structures behind the TM [24]. Results showed that contribution of middle ear structures to reflection was larger at higher wavelengths. The authors concluded that the TM is more transparent above 600 nm. Reflection was minimal around 420 nm and another local minimum was found in the range of 540 to 580 nm. These results perfectly match the general absorbance characteristics as found in the current study with lower absorbance / higher transmission at longer wavelengths and the local absorbance maxima at 420 nm and 540/580 nm. The fact that the local peaks indicate less reflection [24] but also less transmission (current study) leads to the assumption that at these wavelengths absorption plays a major role. As these local absorbance maxima were less pronounced in neonatal rats compared to adult rats and human samples, and the amount of tissue and bone that remained at the samples was lower for neonatal TM, these peaks might be caused by blood and / or tissue / bone. Absorbance spectra of hemoglobin show prominent peaks at above 400 nm and at 540/580 nm with oxygenated hemoglobin being responsible for the double peak [25]. Therefore we might conclude that the measured absorbance maxima are caused by remaining blood on the samples or the tissue containing blood cells. This is also supported by the fact that in control experiments with bone and some tissue still being attached to the bone also these peaks were detected.

Resulting spectra were also dependent from the type of scan. The spectral scan measurements are based on one large spot per well containing information from bone, TM, tissue and empty areas. The more specific area scan is limited by the 9x9 matrix with 81 measuring points. Overlapping of these individual measuring points is unavoidable and must be taken into account when looking at the results. Using the area scan approach, a spot with low absorbance surrounded by spots with higher absorbance was detected in the heat maps for all samples. The position of this spot was stable throughout the measurements except when adding PBS to protect the samples from drying. Measured values at the spot of low absorbance were taken as absorbance values of the TM. As the spots are expected to overlap, even these values will be influenced by the surrounding tissue and bone, which most likely leads to an overestimation of the absorbance in all measurements. That the above mentioned absorbance maxima were smaller in area scans compared to spectral scans supports our approach for the measurement of optical TM absorbance, but the appearance of (weak) peaks also shows the mentioned limitations.

The absorbance of the adult TM is significantly higher than in TM from neonatal rats. Preparation of neonatal TMs was much easier compared to the one of adult TMs with firm bone. To avoid damage to the samples, preparations of adult TM contained more bone and appeared more “bloody”. The influence of the remaining surrounding tissue is also reflected by the measured larger peaks in area scans of adult TM. These preparation induced differences might contribute to and explain at least parts of the measured differences in absorbance between neonatal and adult rats. Additionally, also developmental differences could play a role as the hearing system is not yet functioning in rats at p3-5 [22].

Since all measurements were carried out without addition of fluid, the influence of possible drying of the TM had to be investigated during the measurements, whereby no significant differences to the first measurements could be determined. As no differences were found, we may also speculate that reflection at access fluid on the membrane at the beginning (compare Fig 1) does not play a role for the measurement in our setup, as the TM appeared to have a dry surface after the measurements.

The collection and extraction of sufficient large pieces of intact human TM for the measurements were challenging. In the majority of cases where STP is planned/performed, the patients have previously undergone radical and/or reconstructive ear surgery due to chronic OM or recurrent cholesteatoma. Therefore, the four samples of human TM differed in appearance and characteristics (e.g. presence of malleus, thickness or morphology of the TM due to prior ear surgeries), influenced by the etiology of the patients. The samples clearly differ in clarity of the membrane and the surrounding tissue from those of the neonatal rats. In some cases even a clear view on the TM was hard to achieve. This also explains the higher absorbance compared to the scans of the rat TMs, as well as the greater variability between the scans. The position of the peaks did not change and all scans have the same general properties as the rat TM. Only the third human sample showed a clear difference of the second spectral scan (done after the area scan) to the first spectral scan (before the area scan). This patient had a history of reconstructive ear surgery due to recurrent cholesteatoma. We can only speculate that this earlier reconstruction plays a role in this observation.

All together and due to the above discussed reasons, we expect the absorbance of the TM being slightly lower than measured in the current setting. Whether the amount of light being transmitted through the TM would be sufficient to induce aPDT remains to be tested in further experiments. A further possible application could be the use of high-energy visible (HEV) light (400–450 nm) to kill bacteria as recently shown in vitro for bacteria involved in otitis media [26]. These authors concluded that the appropriate dosage of light would be a combination of the intensity and the duration of exposure whereby less intensity requires more time for inactivation.

## Supporting information

S1 Fig Area scans of five TM. Scans were performed directly after preparation (solid lines) and after 2 hours (dotted lines) from 300 to 900 nm. No effects of drying were detected without additional wetting for at least 2 hours.
(TIF)

S2 Fig Spectral scan of human sample 3. Scans were performed before (red) and after (blue) the area scan with about 1 hour in between.
(TIF)
