# Supplementary figures and images for: Optical absorbance of the tympanic membrane in rat and human samples
Source: PLoS One. 2021 Jul 22;16(7):e0254902. doi: 10.1371/journal.pone.0254902 (PMC8297804; doi:10.1371/journal.pone.0254902)

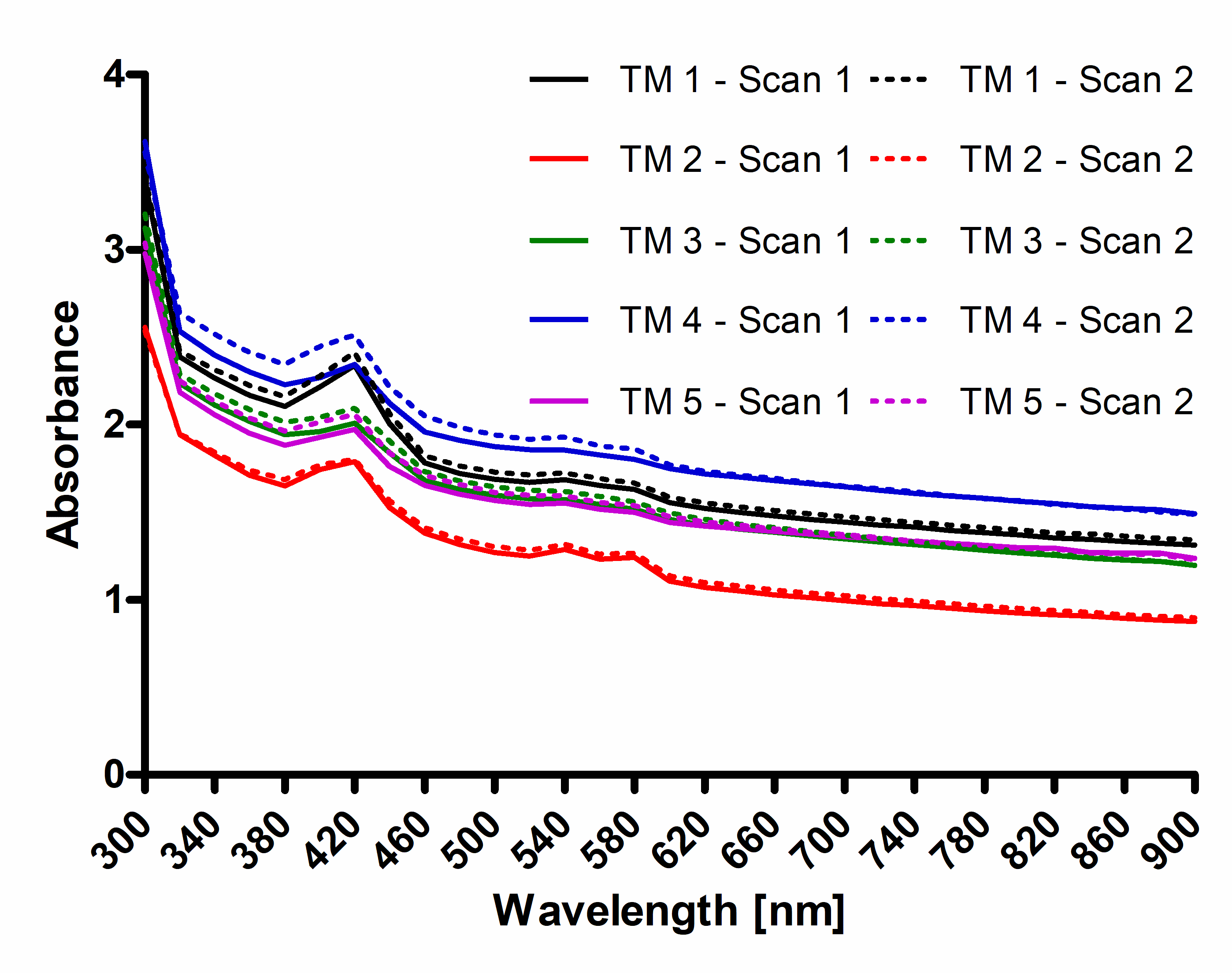

Supplement: S1 Fig — Scans were performed directly after preparation (solid lines) and after 2 hours (dotted lines) from 300 to 900 nm. No effects of drying were detected without additional wetting for at least 2 hours. (TIF) [file pone.0254902.s001.tif]

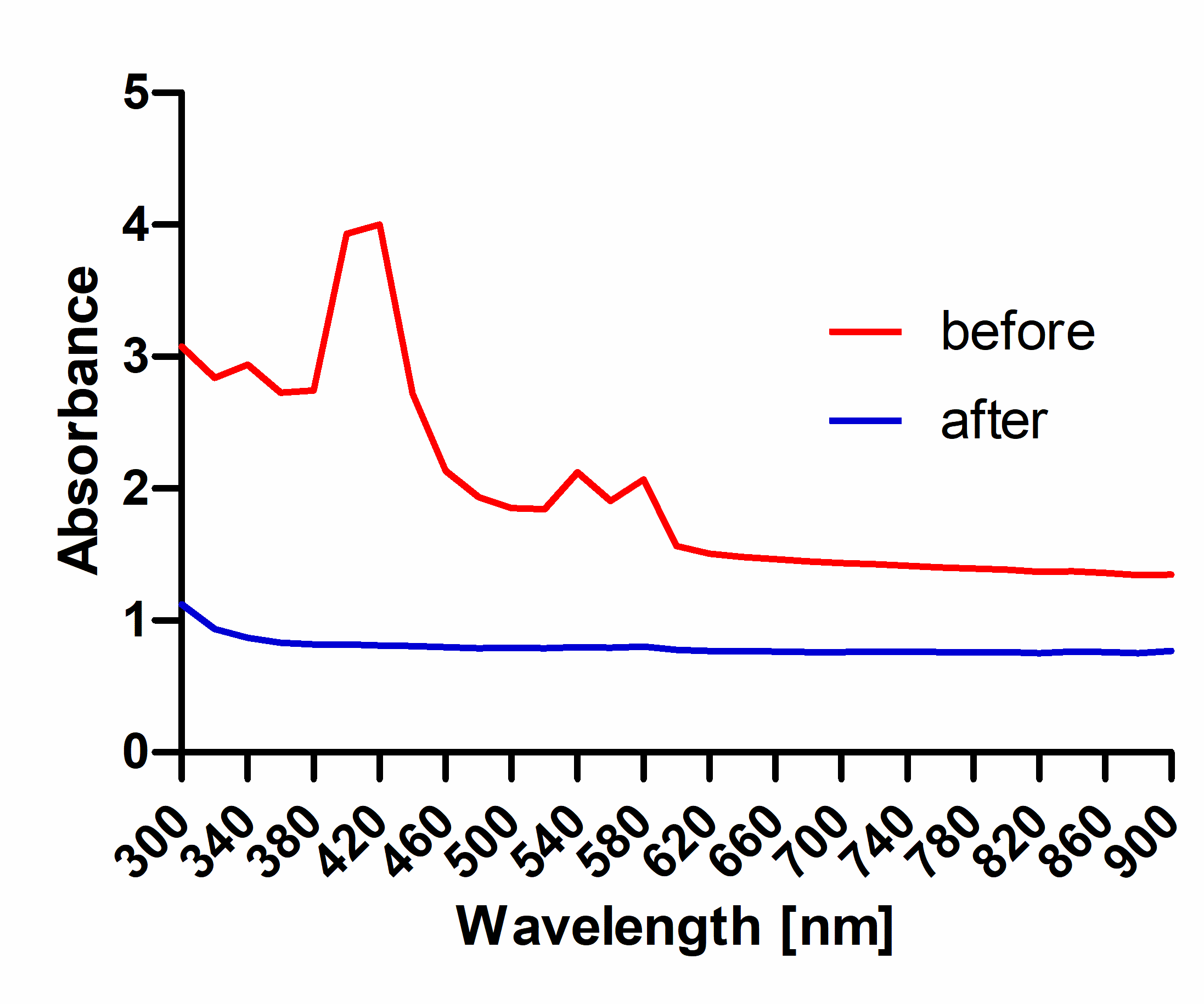

Supplement: S2 Fig — Scans were performed before (red) and after (blue) the area scan with about 1 hour in between. (TIF) [file pone.0254902.s002.tif]
